# Den‐Site Behavior of Bengal Foxes (*Vulpes bengalensis*) Reveals Persistent Use, Social Interactions, and Coexistence in Shared Spaces

**DOI:** 10.1002/ece3.73371

**Published:** 2026-04-02

**Authors:** Muntasir Akash, Md. Rokonuzzaman, Sultan Ahmed, Mohammad Samiul Alam, Maximilian L. Allen

**Affiliations:** ^1^ Department of Zoology University of Dhaka Dhaka Bangladesh; ^2^ Illinois Natural History Survey, Prairie Research Institute University of Illinois Champaign Illinois USA

**Keywords:** camera trap, *Canidae*, den, interspecific interaction, parental care, site fidelity

## Abstract

The Bengal fox ( *Vulpes bengalensis* ) is a small, den‐obligate, wild canid that faces growing pressures in non‐protected landscapes. In Bangladesh, the species is considered vulnerable, receives minimal conservation attention, and knowledge of many components of its ecology is lacking. We present a fine‐scale behavioral account of the Bengal fox at a den site, based on 12.18 h of video recorded during camera trapping from March 2024 to May 2024 in a peri‐urban landscape of north‐western Bangladesh. Our analyses indicated high site fidelity and consistent crepuscular den‐site activity in Bengal foxes. Our estimated lorelograms indicated that foxes remained active at the den site for up to 2 h following an initial detection, confirming behavioral persistence around the den. Fox activity declined gradually with the progression of the survey period, coinciding with the breeding cycle. Behaviorally, foxes engaged in structured, non‐random behavior, with routine bouts of foraging, feeding, grooming, and vigilance as well as complex affiliative social interactions. Foxes displayed persistent antagonistic interactions with Bengal monitors ( *Varanus bengalensis* ), but their avoidance of humans and feral dogs appeared temporal, not spatial. We noted insects, birds, and rodents in feeding activity, including foraging on termite swarms. These findings suggest that Bengal foxes persist in shared spaces and highlight the necessity of quantifying the potential risks of living with dominant predators in human‐dominated landscapes. Although constrained by observations from a single natal den, we provide evidence to consider the species in the mainstream conservation dialog by integrating behavioral data from camera traps with fine‐scale modeling. We further offer a replicable methodological framework for behavioral studies of denning carnivores—especially across the Global South, where data deficiency often hinders policy and action.

## Graphical abstract

We documented fine‐scale denning behavior of the Bengal fox ( *Vulpes bengalensis* ) using 12.18 h of camera trap video from a peri‐urban landscape in north‐western Bangladesh. Analyses revealed strong site fidelity, crepuscular activity, and structured behavioral patterns including foraging, grooming, vigilance, and social interactions. Our findings highlight the species' adaptability to human‐dominated landscapes and present a replicable camera trapping framework and analytical approaches for studying denning carnivores in data‐deficient regions of the Global South.

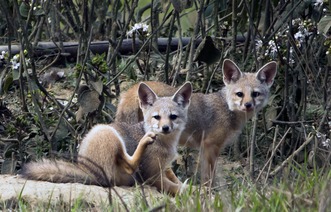

## 1 Introduction

Shelter is a key component of the natural history of animals because it directly affects survival, and hence population persistence and growth (Kinlaw 1999). One of the predominant forms of shelter is the use of natal dens (Johnsingh 1978)—excavated or adopted structures which protect neonates from predators and environmental extremes (Yovovich et al. 2020; Naderi et al. 2024) while providing access to resources required to raise young (Allen and Moll 2023). These factors contribute to the breeding success in denning species (Szor et al. 2008; Yovovich et al. 2020). However, natal dens—found across varied habitats, including human‐modified landscapes—and den‐related behaviors shape intra‐ and inter‐specific interactions (e.g., Tannerfeldt et al. 2003; Scheinin et al. 2006; Rodnikova et al. 2011; Eid and Alayyan 2025). Understanding denning ecology is therefore key to conservation efforts (Yovovich et al. 2020; Naderi et al. 2024; Allen et al. 2025), particularly for species that are reliant on specialized den sites amid accelerating habitat loss, landscape modification and climate change.

Among carnivores, canids are widely known for using dens for breeding and rearing pups. Canid dens may be self‐excavated holes in the ground, or repurposed from burrows, tree hollows or rocky crevices (Mukherjee et al. 2018; Allen and Kritzer 2023). Canid activity around dens varies seasonally, typically synchronized with the breeding calendar so that most activity is concentrated in spring and summer while rearing young (Home and Jhala 2010; Allen and Moll 2023). Natal denning also reflects strong pair bonds and complex social interactions, entailing central place foraging strategies to maximize reproductive success (Allen and Moll 2023; Allen and Kritzer 2023). While studies on canid denning ecology are well‐represented in temperate regions, comparable research in tropical contexts—particularly in South Asia—remains limited (but see Punjabi et al. 2013; Mukherjee et al. 2018; Iqbal et al. 2025).

**FIGURE 1:**
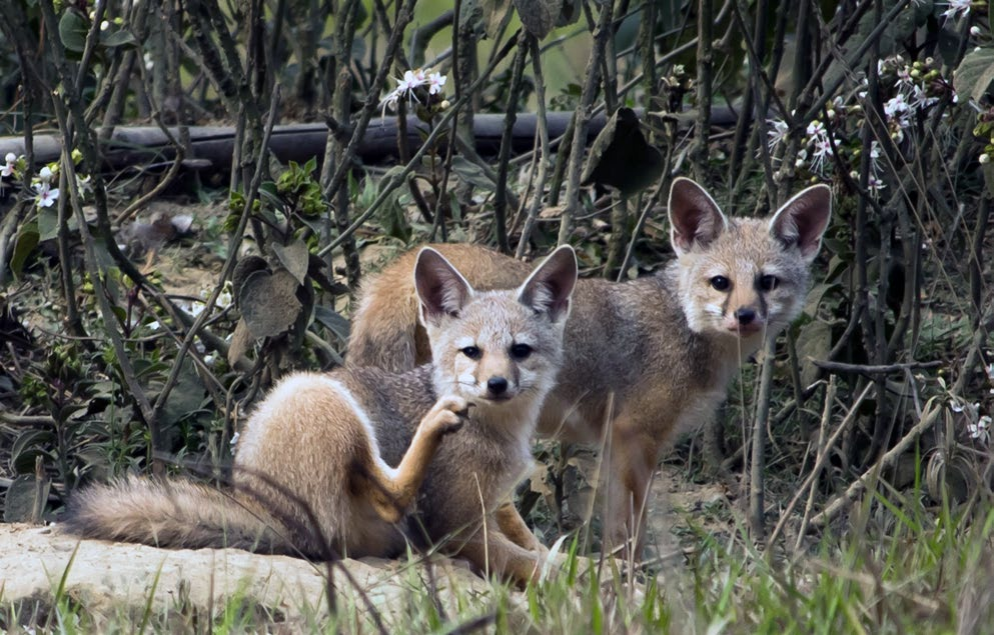
Bengal foxes ( *Vulpes bengalensis* ) at the monitored den site in north‐western Bangladesh, photographed during the survey period (March–May 2024). Photo credit: Sultan Ahmed.

**FIGURE 2:**
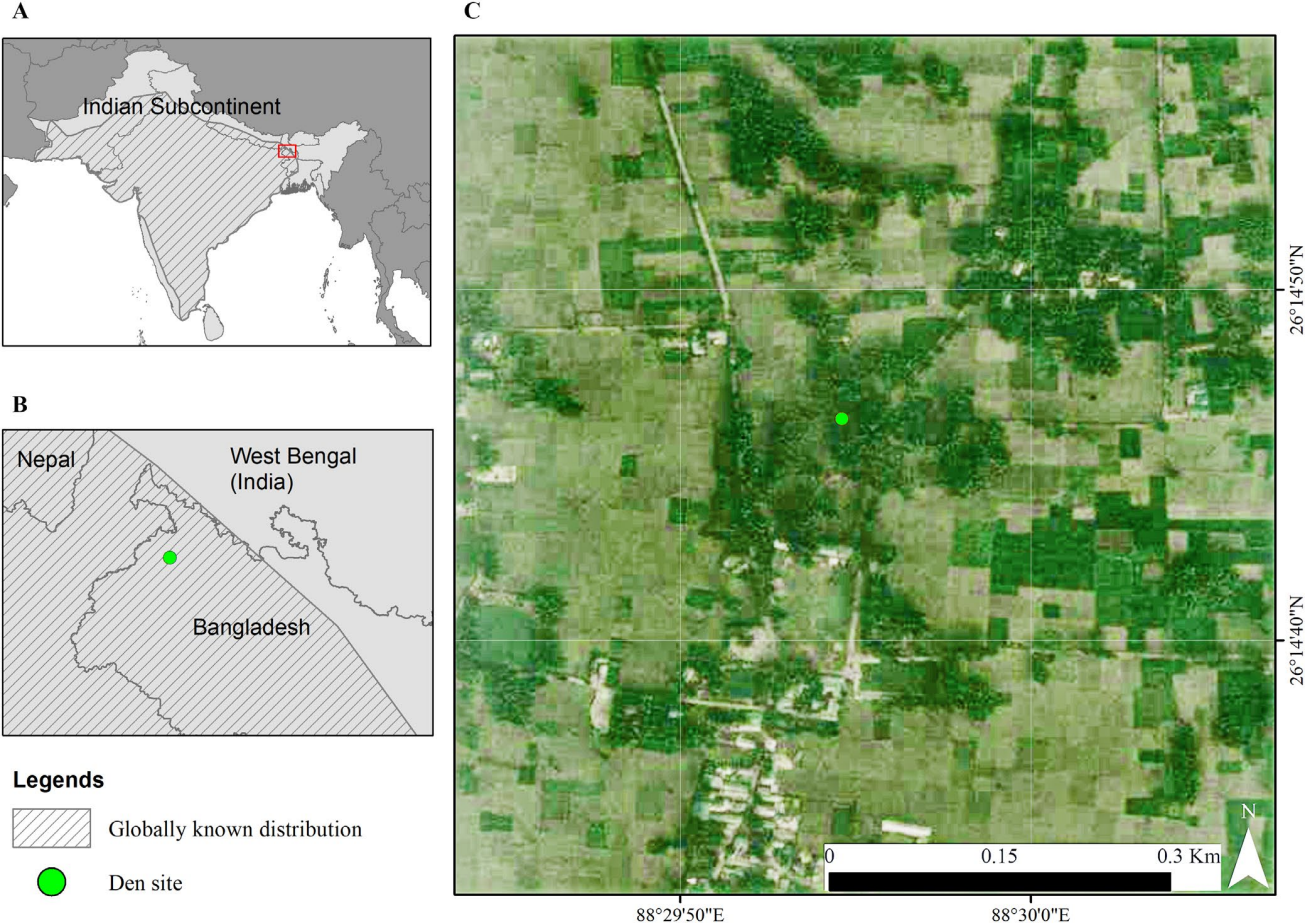
(A) Distribution range of the Bengal fox ( *Vulpes bengalensis* ), according to the latest IUCN global assessment (Jhala 2016), (B) the location of the den site we monitored with a camera trap in north‐western Bangladesh, and (C) overview of the habitat at the camera trapped den site, using 2024 ESRI World Imagery Map as a background (*Source:* ESRI, Maxar, GeoEye, Earthstar Geographics).

The Bengal fox ( *Vulpes bengalensis* ) is a den‐obligate, small canid, endemic to the Indian subcontinent (Figure 1). Bengal foxes are monogamous, with a single breeding pair sharing a natal den during the pup‐rearing season. The male actively participates in pup‐rearing, including provisioning and den guarding, and collective denning has not been reported (Johnsingh 1978; Gompper and Vanak 2006). The species is ecologically flexible but poorly understood and increasingly threatened. For example, its den site preferences vary across studies: Vanak and Gompper (2010) reported an affinity to native grasslands; however, Punjabi et al. (2013) observed no avoidance of agricultural landscapes—and both studies were conducted in the same site in Maharashtra, India. While the species is considered a habitat generalist, global assessments highlight its grassland dependence (Jhala 2016). Yet, studies from eastern India (e.g., Johnsingh 1978; Das et al. 2022) and northwestern Bangladesh (Akter et al. 2023) documented foxes in shared spaces. The Bengal fox is considered a species of Least Concern according to the IUCN (Jhala 2016), and thus garners little conservation attention (Vanak et al. 2008). Foxes' populations are declining due to habitat loss and land‐use change (Home and Jhala 2009; Vanak and Gompper 2009). The species is facing regional extirpation, with only about 1% of its predicted distribution falling within protected areas (Vanak et al. 2008; Jhala 2016; Desai et al. 2023). In Bangladesh, the species is Vulnerable, now appearing confined to a reduced range west of the Brahmaputra River (Figure 2; Khan 2015; Jhala 2016), with only one prior study to date (Akter et al. 2023).

Aspects of the Bengal fox's denning and diet have been described primarily from central and southern India. An omnivorous and opportunistic feeder with an invertebrate‐heavy diet, Bengal foxes are primarily nocturnal and crepuscular (Gompper and Vanak 2006; Jhala 2016; Vanak and Gompper 2009; Desai and Dharaiya 2023; Akter et al. 2023; Naderi 2025). Dens are essential for reproduction, with foxes exhibiting strong pair fidelity and excavating multi‐entrance burrows to rear kits (Johnsingh 1978; Kumara and Singh 2012; Castelló 2018; Niraula et al. 2020; Akter et al. 2023). Some studies documented behavior through direct observation (Dookia et al. 2012) and estimated density via breeding pair counts (Home and Jhala 2010). However, recent findings of ophiophagy (Desai et al. 2022), den sharing with monitor lizards ( *Varanus bengalensis* ) (Desai et al. 2021), and range expansion into northeast India (Choudhury 2020) reflect the species' poorly known status.

Camera traps are reliable and cost‐effective non‐invasive tools for behavioral studies (Bridges and Noss 2011; Caravaggi et al. 2017; Adewale et al. 2022; Bhardwaj et al. 2022). Yet a review by Burton et al. (2015) found only 10 camera trap studies from South and Southeast Asia that addressed behavioral questions. The limited application of camera traps to understand South Asian canid denning ecology is likely shaped by several persistent challenges: low detection probabilities due to target species' elusiveness, low‐density populations, and potential behavioral avoidance of camera traps (e.g., see Séquin et al. 2003; Vanak and Gompper 2007; Mortelliti et al. 2024). High rates of theft and vandalism—especially in human‐dominated landscapes—add to the challenge (Meek et al. 2019). Low conservation investment in lesser‐known species like the Bengal fox (see Vanak et al. 2008) additionally restricts robust studies—a common scenario in the Global South (see Srivathsa et al. 2022). Although Vanak and Gompper (2007) recommended camera trapping as an effective tool to study Bengal foxes, such efforts are rare, and the species' response to camera traps remains unquantified. Moreover, fine‐scale behavioral patterns—particularly activity and interactions near dens—are lacking, unlike in other canids (e.g., Allen and Kritzer 2023).

In this study, we used camera traps in a human‐dominated landscape of north‐western Bangladesh from March 2024 to May 2024 to understand Bengal fox behavior at their den. We considered four hypotheses: (1) Bengal fox activity at the den‐site would remain concentrated until the completion of the breeding cycle in May 2024; (2) repeated camera trap checks by researchers would negatively affect fox visitation rate at the den site; (3) Bengal foxes would exhibit temporal segregation from potential threats (i.e., more dominant carnivores and humans); and, (4) camera‐trapping would reveal foxes' behavioral association at intra‐ and inter‐species level.

## 2 Materials and Methods

### 2.1 Study Area

We monitored a Bengal fox den in an unfenced graveyard (26.2462° N 88.4985° E) in Panchagarh District, north‐western Bangladesh, which shares a border with India (Figure 2). The landscape belongs to the Old Himalayan Piedmont Plain at 70 m a.s.l. elevation with declining precipitation (about 8.8 mm per century) (Akter et al. 2021). Its subtropical climate has distinct dry season (November–May, 10°C–20°C, 1580–2000 mm) and monsoon (June–October, 25°C–38.5°C, 2600–3200 mm) (Nishat et al. 2002; Akter et al. 2021). Having no protected area (IUCN Bangladesh 2015), anthropogenic footprints dominate the district, comprising a network of peri‐urban and rural settlements, cultivated fields, tea gardens, orchards and agroforests, and stone quarries along riparian, transboundary rivers. Dominant flora surrounding the den site include the mango ( *Mangifera indica* ), tamarind ( *Tamarindus indica* ), teak ( *Tectona grandis* ), eucalyptus (*Eucalyptus* sp.) and mahogany ( *Swietenia mahagoni* ), with perennial and ephemeral shrubs as undergrowth on well‐drained, sandy‐loamy soil. Common extant terrestrial mammals in the area are typical of the region, including the golden jackal ( *Canis aureus* ), jungle cat ( *Felis chaus* ), fishing cat ( *Prionailurus viverrinus* ), small Indian mongoose (*Urva auropunctata*), South Asian civets (*Viverricula, Viverra*, and *Paradoxurus*), black‐naped hare ( *Lepus nigricollis* ), and various murid rats (IUCN Bangladesh 2015).

### 2.2 Data Collection and Processing

In early March 2024, we found the den site as part of a wider survey on Bengal fox distribution and ecology in Bangladesh. After observing the den site being actively used by foxes with the presence of kits, we deployed a single camera trap to monitor their activity and that of other species at the den site. We monitored the den continuously from 08 March 2024 until 28 May 2024, with brief gaps due to depleted batteries and camera malfunctions. We used two different models of camera traps with a field of view of about 5 m wide by 2.5 m high, starting with a Browning Dark Ops Apex (Model no. BTC‐6HD‐Apex, Birmingham, AL, USA). After we found the camera trap malfunctioning on 18 March 2024, we used a Bushnell Prime Low Glow (Model no. 119932C, Overland Park, KS, USA) for the remaining period. We programmed the cameras to record a 10‐s video with a 10‐s delay between triggers and changed batteries and checked memory cards about every 2 weeks. We mounted the camera trap on a eucalyptus, about 70 cm off the ground. We directed the angle of view downward ∼40° relative to the vertical axis, about 3 m away, and centered on the main and largest of the six openings we spotted; however, the internal den structure was unknown.

We carried out data curation, metadata extraction, and all following statistical analyses in the program R (version 4.4.1; R Core Team 2024), and for each statistical test, we set alpha at 0.05. First, we identified vertebrates to the species level, including anthropogenic disturbance (human and feral dog movement but we used researchers' activity detected by the camera trap as a separate detection variable), that were detected in video clips recorded by the camera trap, and subsequently tagged the clip metadata using a digital asset management application (Adobe Bridge, Adobe Inc., San Jose, CA, USA). We discarded videos where the species' identity was indiscernible. Then, with the same metadata tagging approach, for each video clip that detected fox movement, we organized them into four broad classes based on the recorded movement characteristics: (1) solitary behavior, exhibited by a single individual in the absence of visible interaction with conspecifics or other species; (2) inter‐species interaction, behaviors occurred between two or more species; (3) intra‐species interaction, behavioral responses of the Bengal fox toward other individuals; and (4) undefined behavior, video clips where fox was detected but behavioral pattern was not definable (e.g., due to partial visibility, short duration, or indistinct movement) for confident classification. For the first three classes, we further sorted video clips into different behavioral categories, respectively. We provide an ethogram with their operational definitions (Table 1), which we developed inductively.

**TABLE 1:** An ethogram of different behaviors exhibited by the Bengal fox and detected at the den site camera‐trapping survey in north‐western Bangladesh from 08 March 2024 to 28 May 2024.

Behavioral types	Description
Solitary behavior	
Autogrooming	The Bengal fox cleans and maintains its own fur through licking, biting, or scratching
Caching food	The Bengal fox stores food items by burying them
Defecating	The Bengal fox defecates and then drags its hindquarters along the ground, likely to mark territory or maintain hygiene
Feeding	The Bengal fox actively consumes an identifiable or unidentifiable food item
Foraging	The Bengal fox exhibits exploratory movements such as bobbing its head with an extended neck near the ground to detect cues—often followed by digging—and/or surveying the surroundings through directional head movements, either while stationary or in motion
Passing	A lack of behavior, the Bengal fox just passes through the camera trap's field of view without displaying any other distinct behavior
Resting	The Bengal fox sits motionless on the ground with belly and all four legs tucked in, or lays down on the flank end with front paws relaxed or fully extended
Vigilance	The Bengal fox examines the surroundings in a state of alertness or heightened awareness. The head is fixed directionally, or moved rapidly tracking a stimulus. Individuals are positioned low to the ground with legs splayed and ears held close to the head
Intra‐species interaction	
Allogrooming	The Bengal fox engages in mutualistic fur cleaning and maintenance with another individual through cooperative licking, biting, or scratching
Greeting	The Bengal fox initiates close contact, typically involving sniffing, tail wagging, making low‐pitched sounds, crouching and quivering hindquarters, and brief physical interactions such as rubbing, as part of conspecific recognition or social bonding
Mounting	The Bengal fox mounts another individual in a pseudo‐copulatory manner, often interpreted as a form of social or dominance‐related behavior
Nursing	The Bengal fox offspring gets milk nourishment from mother
Playing	The Bengal fox exhibits conspecific communication involving chasing, counter‐chasing, pouncing, or mock fighting in a non‐agonistic pattern, as well as plays involving manipulation of food and non‐food items
Inter‐species interaction	
Chasing	The Bengal fox pursues another species, often in response to perceived threat or prey
Flight	The Bengal fox flees rapidly from another species, typically as an antipredator or conflict‐avoidance response
Stand‐off	The Bengal fox and another species engage in an interaction marked by close proximity, alert posture, and mutual observation, without immediate aggression or retreat

In cases where we detected multiple behaviors within a single video clip, we tagged all relevant behaviors. We defined a behavioral bout as a discrete period of activity separated by inactivity, ending when foxes left the camera frame or the behavior was discontinued. We marked such start and end points and counted the number of behavioral bouts.

We analyzed modularity patterns at the level of discrete behavioral bouts to ensure that we did not inflate co‐detection due to prolonged activity; however, this unit was not always appropriate for rarer behaviors. In particular, inter‐species and intra‐species interactions, as well as feeding events, are comparatively infrequent and often visually distinct even in short clips. To retain the full behavioral variation and maximize taxonomic and interactional resolution, we used all behaviorally classified clips for these specific analyses, including those not assigned to discrete bouts.

We counted the maximum number of foxes detected in each video clip. However, we could often not identify the sexes and age classes as the Bengal fox is an unmarked species (Vanak and Gompper 2007) and, in the latter period of camera trapping, age differentiation became unclear. In case of any bout of inter‐species interaction, we also counted the number of individuals of the other species involved.

In case of invertebrates detected, or whenever foxes were observed carrying items, we tagged these under general groups (i.e., insects, termite swarms, rodents, birds, and unidentified items), because video quality did not allow for a higher‐level identification. Then, we extracted the date, time, and duration of visits for each species and fox behaviors with the package *camtrapR* (Niedballa et al. 2016) and calculated summary statistics. For each species, we also calculated relative abundance as the number of video clips per trap night.

### 2.3 Statistical Analyses

We assessed fox responses to anthropogenic stimuli in a two‐step approach. First, using the package *mgcv* (Wood 2017), we fitted generalized additive models (GAM) with counts of fox video clips for each camera trap day as a proxy of fox activity level. Our global model included five different predictors: Julian day, camera trap check, days since last check, and daily video clip counts of the next two most frequently detected species—Bengal monitor ( *Varanus bengalensis* ) detections and human movement detections. We considered Julian day as a proxy of seasonal variation. Camera trap check, a binary variable for each camera trap day, specified days when researchers visited the site for extracting data. Days since last check referred to the number of days since the last camera trap checking, which we included to capture any lingering effects.

We considered the potential impacts of Julian day and days since last camera trap check to be non‐linear and selected a cyclic regression spline for both. We considered our hypothesized a priori predictors to have negative effects on fox activity (Table S1). Because of overdispersion, confirmed via diagnostic checks, we followed a negative binomial framework (Zuur et al. 2009). No continuous predictors appeared confounded after screenings for multicollinearity (Zuur and Ieno 2016). For relative model selection, we selected the most parsimonious model, considering the lowest Akaike Information Criterion (AIC) values and comparing nested models with the likelihood ratio test (Zuur et al. 2009). We did not use any shrinkage or smoothing parameters. Checks for the absolute fitness of all models followed Zuur et al. (2009). We conducted 500 bootstrap simulations and checked the approximation of the effective degrees of freedom (edf) to the number of iterations (*k*′) to assess models' ability to adequately estimate the wiggling effect. In additive models, a low *p* value (< 0.05) associated with *k*‐index (< 1) suggests a potential goodness of fit issue, that the chosen iteration for smoothing parameters is potentially low (Wood 2017).

Secondly, we used the package *lorelogram* (Iannarilli et al. 2019) to assess the short‐term temporal dependency in fox activity at the den site. Lorelograms identify dependence structure in temporally replicated dataset. The approach can guide robust aggregation of independent camera trap detections of wildlife. This is advantageous in many cases, including kernel density estimates of diel activity—which generally relies on an arbitrary temporal threshold to consider camera‐trap detections as independent. Lorelograms visualize correlation structures in binary data by plotting pairwise log‐odds ratios (*ψ*) of binary outcomes *Y* versus time (*t*) incremented by △*t* time units, such that:

$$\log|\psi \left(Y_{t},Y_{t+\Delta t}\right)|=\log\left|\frac{P\left(Y_{t+\Delta t}=1|Y_{t}=1\right)\cdot P\left(Y_{t+\Delta t}=0|Y_{t}=0\right)}{P\left(Y_{t+\Delta t}=0|Y_{t}=1\right)\cdot P\left(Y_{t+\Delta t}=1|Y_{t}=0\right)}\right|$$

where 1 and 0 s refer to detections and non‐detections at △*t* intervals (Heagerty and Zeger 1998; Iannarilli et al. 2019).

When considering the den site fidelity of Bengal foxes during the breeding season, we hypothesized that the correlation structure in foxes' short‐time temporal dependence would not be zero or negative, but only decay with increasing time lags. We also hypothesized that the presence of camera traps and the periodic camera checks would not disrupt this temporal structure, as both conditional events are reported to develop camera trap avoidance in carnivore mammals (see Tourani et al. 2020). To assess this, we examined the time point at which the correlation structure decayed by determining when the first derivative of the log‐odds ratio function relevant to △*t* approached zero. Assuming no impact, we also considered that this decay pattern would not vary across six discrete sessions, each separated by a camera trap checking event. Following Iannarilli et al. (2019), we prepared a fox detection history at a 1‐min scale and visualized lorelograms up to a maximum time lag of 360 min, both for the entire sampling period and six discrete sessions—each separated by a preceding check of the camera trap.

To understand the diel activity pattern of foxes at the den site and the extent of temporal partitioning with other species visiting the den site, we conducted three different sets of analyses. Prior to analyses, we converted all clock time to circular radian format and classified detections into three different diel periods following the positions of the Sun's center at the horizon: twilight (detections occurring 1 h before and after both sunrise and sunset); daytime (detections occurring > 1 h after sunrise and < 1 h before sunset); and nighttime (detections occurring > 1 h after sunset and < 1 h before sunrise). Here, we used the packages *lubridate* (Grolemund and Wickham 2011), *astroFns* (Harris 2019), and *suncalc* (Thieurmel and Elmarhraoui 2022). The following analyses used the *activity* (Rowcliffe 2021), *overlap* (Meredith and Ridout 2014), and *Diel.Niche* (Gerber et al. 2024) packages.

For the Bengal fox and other species with ≥ 20 detections (kernel density estimates can become unreliable below this threshold, see Ridout and Linkie 2009), we first assessed kernel density estimates to visualize diel activity distributions and compared pairwise overlap between activity distributions. For each comparison, we calculated the coefficient of overlap (Δ) which ranges from 0 (no overlap) to 1 (identical temporal activity) and 95% confidence intervals (CI; based on 10,000 empirical bootstrapped resamples) to obtain a relative measure of variation across comparisons. Following Ridout and Linkie (2009), we used Δ_4_ when both species had > 75 detections; otherwise Δ_1_, as recommended for small samples.

Using the *Diel.Niche* package (Gerber et al. 2024), we estimated quantitative definitions of diel phenotypes exhibited by foxes at the den site. Based on the number of detections during daytime, nighttime, and crepuscular hours, we estimated Bayesian posterior probabilities and 95% Bayesian credible intervals (BCI) to evaluate two hypotheses proposed by Gerber et al. (2024). First, we tested whether foxes follow a strategy of maximizing activity during a specific period (i.e., which diel phase is used most). Second, we assessed time‐period selection by comparing the proportion of detections (“use”) within a given diel phase to that phase's proportional “availability” based on the length of the diel cycle. For both analyses, we used three Markov chains with 5000 iterations each, discarding the first 1000 iterations as burn‐in and ensuring convergence before inference.

We constructed a network structure to visualize the association between different behaviors exhibited by foxes at the den site. We split behaviors into individual terms when multiple behaviors were detected within a single bout (e.g., foraging followed by resting), extracting all possible pairwise combinations. To reduce noise from rare detections, we excluded infrequent behaviors, applied a threshold, and retained only behavioral pairs with ≥ 10 co‐occurrences (see Vázquez et al. 2007; Pinheiro et al. 2019).

We constructed an adjacency matrix from these co‐occurring behaviors, summing symmetrical connections to yield a weighted, undirected matrix. In the network, nodes represented distinct behavioral categories, and edges indicated co‐occurrence counts between two behaviors within a single video clip.

To test whether the observed network structure exhibited significant modularity (i.e., the presence of non‐random clusters of co‐occurring behaviors), we used the *bipartite* package (Dormann et al. 2008). The observed modularity was assessed against 1000 null models generated with the Vázquez null model (see Vázquez et al. 2007; Pinheiro et al. 2019), which preserves marginal totals of the observed matrix while randomizing link distributions. We assessed significance with a one‐tailed test comparing the observed modularity to the null distribution.

## 3 Results

### 3.1 Species Observed

**FIGURE 3:**
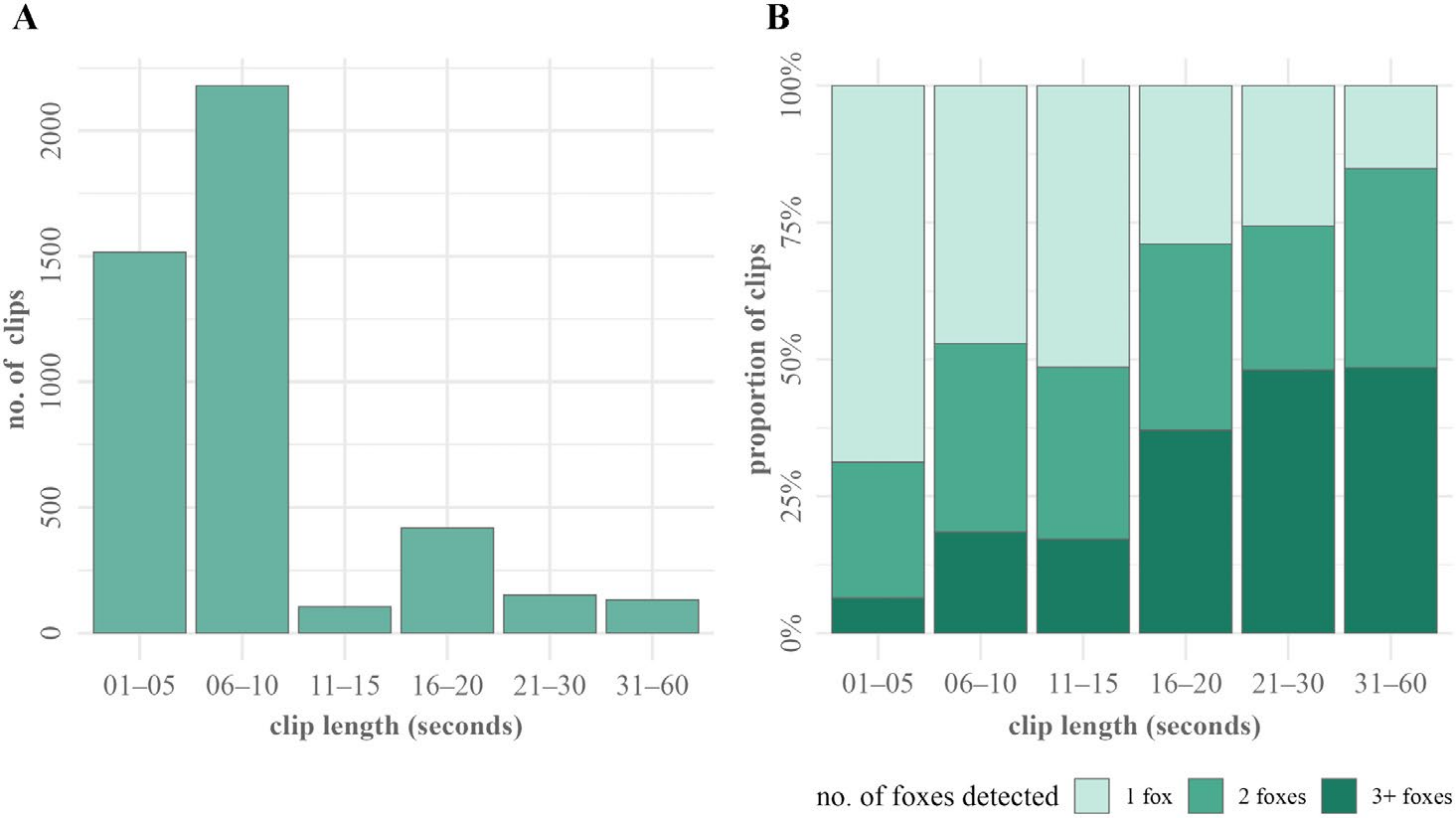
Distribution of camera‐trap video clips by clip length (seconds) recorded at the den site survey in north‐western Bangladesh from 08 March 2024 to 28 May 2024 (A), and the proportion of clips within each clip‐length bin categorized by the number of Bengal foxes ( *Vulpes bengalensis* ) detected (B).

Across 58 active camera trap days at the den site, we recorded 12.18 h of video comprising 4641 video clips, and documented 10 different vertebrate species (2 carnivores, 1 reptile, and 7 birds) and two types of human‐induced activity (human movement and feral dog movement, Table 2). Mean clip length was 9.45 s (SD ± 8.19 s) (Figure 3A). Bengal foxes appeared in 4502 of these clips, including 4053 single‐species detections and 449 co‐detections with two different species (Bengal monitor = 447, jungle babbler = 2), totaling 11.75 h of video (Table 2). Bengal fox detections declined steadily from March (*n* = 2758; 18 camera trap days) to April (1510; 20 days) and dropped sharply in May (234; 20 days) (Table 2).

**TABLE 2:** Summary of the Bengal fox activity, the species guild, and anthropogenic impacts detected at the den site camera‐trapping survey in north‐western Bangladesh from 8 March 2024 to 28 May 2024.

	Total detections							RAI
	(*n*)	D	N	C	Mar	Apr	May	
Single species detections								
Bengal fox *Vulpes bengalensis*	4053	870	1449	1734	2647	1196	210	69.88
Bengal monitor *Varanus bengalensis*	67	60	1	6	4	54	9	1.16
Human movement	34	30	2	2	8	2	24	0.59
Asiatic golden jackal *Canis aureus*	6	1	2	3	4	1	1	0.10
Feral dog movement	5	2	—	3	4	1	—	0.09
Jungle babbler *Turdoides striata*	17	16	—	1	12	1	4	0.29
Jungle myna *Acridotheres fuscus*	1	1	—	—	—	1	—	0.02
Pied myna *Sturnus contra*	4	4	—	—	—	—	4	0.07
Spotted dove *Spilopelia chinensis*	2	2	—	—	1	—	1	0.02
Gray‐headed woodpecker *Picus canus*	1	1	—	—	1	—	—	0.03
Oriental magpie‐robin *Copsychus saularis*	1	1	—	—	—	1	—	0.02
Common tailorbird *Orthotomus sutorius*	1	—	—	—	1	—	—	0.02
Co‐detections								
Bengal fox and Bengal monitor	447	388	—	59	109	314	24	7.71
Bengal fox and Jungle babbler	2	—	—	2	2	—	—	0.03

*Note:* Relative Abundance Index (RAI) shown for the complete survey period, as well as the number of video clips for the complete survey period (*n*), across three diel periods (Crepuscular, C; Nocturnal, N; Day, D) and three survey months (March, April, and May).

### 3.2 Impact of Disturbance on Den‐Site Activity

**FIGURE 4:**
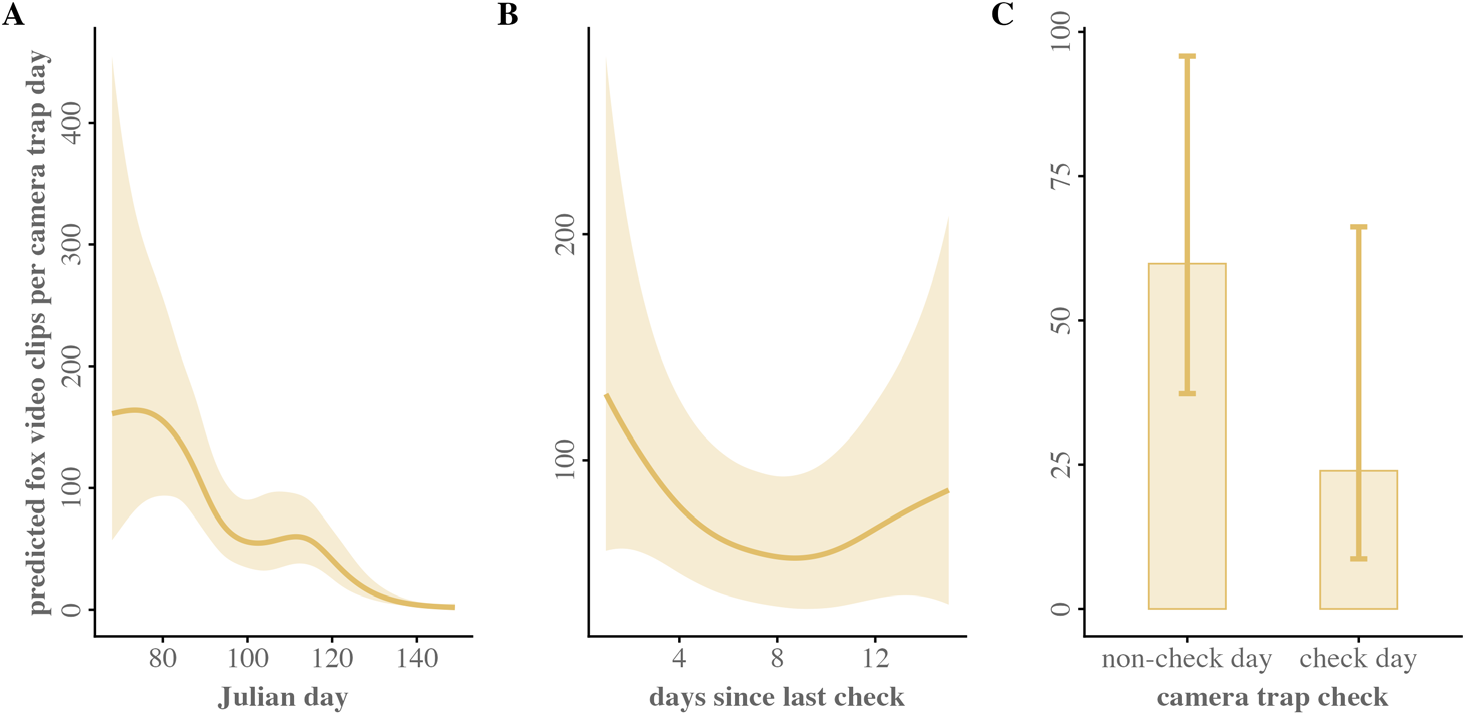
Predicted daily fox video clip counts at the den site as a function of Julian day (A), days since last camera trap check (B), and camera trap check status (C), estimated from the top‐ranked generalized additive model with negative binomial structure. Solid lines denote the predicted smooth function with shades for the 95% confidence interval. The effects shown on each plot are generated by varying the focal predictor while holding all other covariates at their mean values. See Table S2 for full model comparison.

The predicted Bengal fox activity at the den site was best explained by the global model (Table 3, Figure 4; Table S2). The model explained ~50% of variation and significantly outperformed the second‐ranked model (ΔAIC ≥ 1.9), a simpler nested model that included only Julian day (likelihood ratio test, *χ* ^2^ = 16.5, df = 8, *p* = 0.03; Table S2). The predicted fox activity at the den site exhibited strong seasonal variation with a peak around mid‐April, as indicated by the significant smooth term for Julian day (edf = 4.67, *χ* ^2^ = 170.92, *p* < 0.001, Figure 4A). The smooth term for days since last check was estimated with low complexity (edf = 2.21), indicating a near‐linear effect and limited contribution to explaining variation in fox activity at the den site (Table 3, Figure 4B). Of the linear predictors, only the binary predictor, camera trap check (days when cameras were visited for extracting data), exhibited a moderate negative impact on fox activity at the den site (*β* = −0.86, SE = ±0.43, *p* = 0.05; Table 3, Figure 4C). On camera trap check days, the model predicted approximately 60% fewer fox video clips per camera trap day compared to non‐check days (predicted mean: 23.9 clips [95% CI: 8.7–66.2] vs. 59.8 clips [95% CI: 37.3–95.9], respectively), though the wide confidence intervals reflect the relatively few check days in the sampling period (*n* = 6). The effects of Bengal monitor detections and human movement detections were small and non‐significant.

**TABLE 3:** Parametric coefficients and approximate significance of smooth terms of the top‐ranked generalized additive mixed model with negative binomial structure predicting the den site activity of the Bengal fox in north‐western Bangladesh as a smooth function of Julian day and days since last check, and a linear function of camera trap check, Bengal monitor detections and human movement detections.

Predictors	Parametric coefficients				
	Log‐mean	SE	95% confidence interval	*z* score	*p*
Intercept	3.67	0.12	[3.43, 3.91]	−8.76	< 0.001
Camera trap check (Yes)	−0.86	0.43	[−1.70, −0.01]	−1.97	0.05
Bengal monitor detections	0.01	0.01	[−0.01, 0.03]	1.06	0.30
Human movement detections	0.01	0.06	[−0.11, 0.13]	0.21	0.84

Predictors	Significance of smooth terms			
	Effective degree of freedom (*edf*)	Reference degree of freedom (*k*)	*χ* ^2^ value	*p*
Smooth term (Julian day)	4.67	9.0	170.92	< 0.001
Smooth term (days since last check)	2.21	9.0	3.85	0.21

*Note:* The Bengal fox detections per active camera trap day were used as a proxy of the fox activity level at the den site captured in camera trapping from 8 March 2024 to 28 May 2024.

**FIGURE 5:**
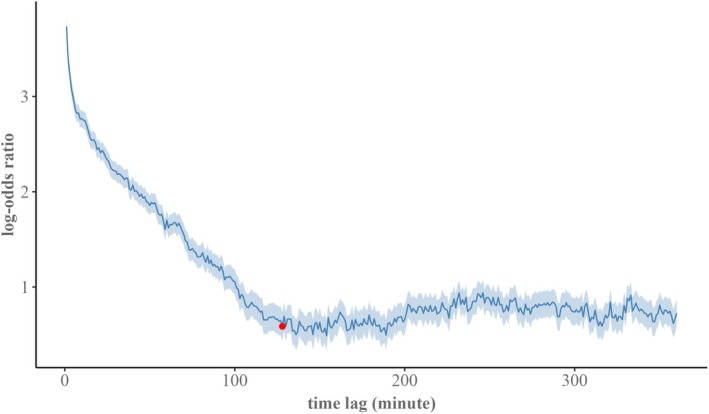
Estimated lorelogram and 95% confidence intervals (shaded areas) for time‐lags between 0 and 360 min for the Bengal fox ( *Vulpes bengalensis* ) activity detected at the den site camera‐trapping survey in north‐western Bangladesh from 8 March 2024 to 28 May 2024. The red circle denotes the time lag when the first derivative of the log‐odds ratio function relative to Δt approximated zero.

The estimated lorelogram for the complete survey period indicated that short‐term temporal dependency in fox activity at the den site declined gradually, with no abrupt subsidence (Figure 5). The foxes appeared to remain at or near the den site for up to 120 min following an initial detection, after which activity reached approximate independence of short‐term autocorrelation as the lorelogram leveled off. When we estimated lorelograms for six discrete sessions, each preceded by a camera trap check, two sessions did not converge, lacking enough detection pairs to reliably estimate log‐odds ratios. For the remaining sessions, temporal dependence in activity became negligible in a similarly gradual pattern, with estimated lag times to reach independence ranging between 168 and 305 min (Figure S1).

### 3.3 Diel Activity Pattern at the Den Site

**FIGURE 6:**
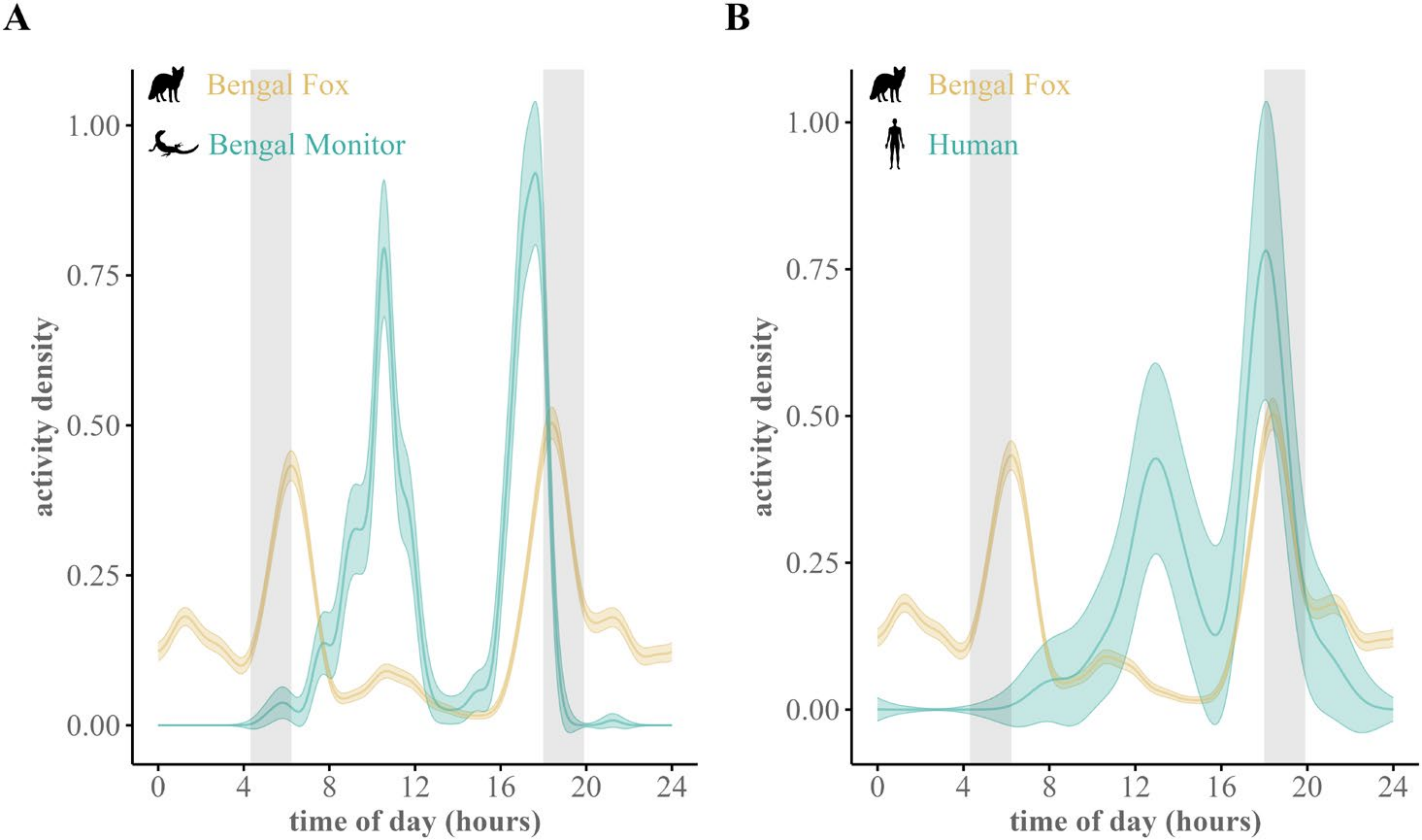
Comparison of the activity pattern of the Bengal fox ( *Vulpes bengalensis* ) detected at the den site camera‐trapping survey in north‐western Bangladesh with (A) that of the Bengal monitor ( *Varanus bengalensis* ) and (B) human movement, summarized for the complete survey period.

Bengal foxes at the den site exhibited primarily crepuscular behavior, with only about 28% of recorded videos being diurnal (Table 2, Figure 6). Fox diel activity peaked during both crepuscular periods (∼430–630 h in the morning and ~1800–2000 h in the evening) but showed moderate intensity during nighttime (Figure 6). Limited sample sizes allowed only two pair‐wise comparisons (Table 2): the temporal overlap between Bengal foxes and Bengal monitors was low (Δ_4_ = 0.26, 95% CI = 0.18–0.35), while Bengal fox overlap with human movement was moderate (Δ_1_ = 0.42, 95% CI = 0.34–0.50) (Figure 6).

**FIGURE 7:**
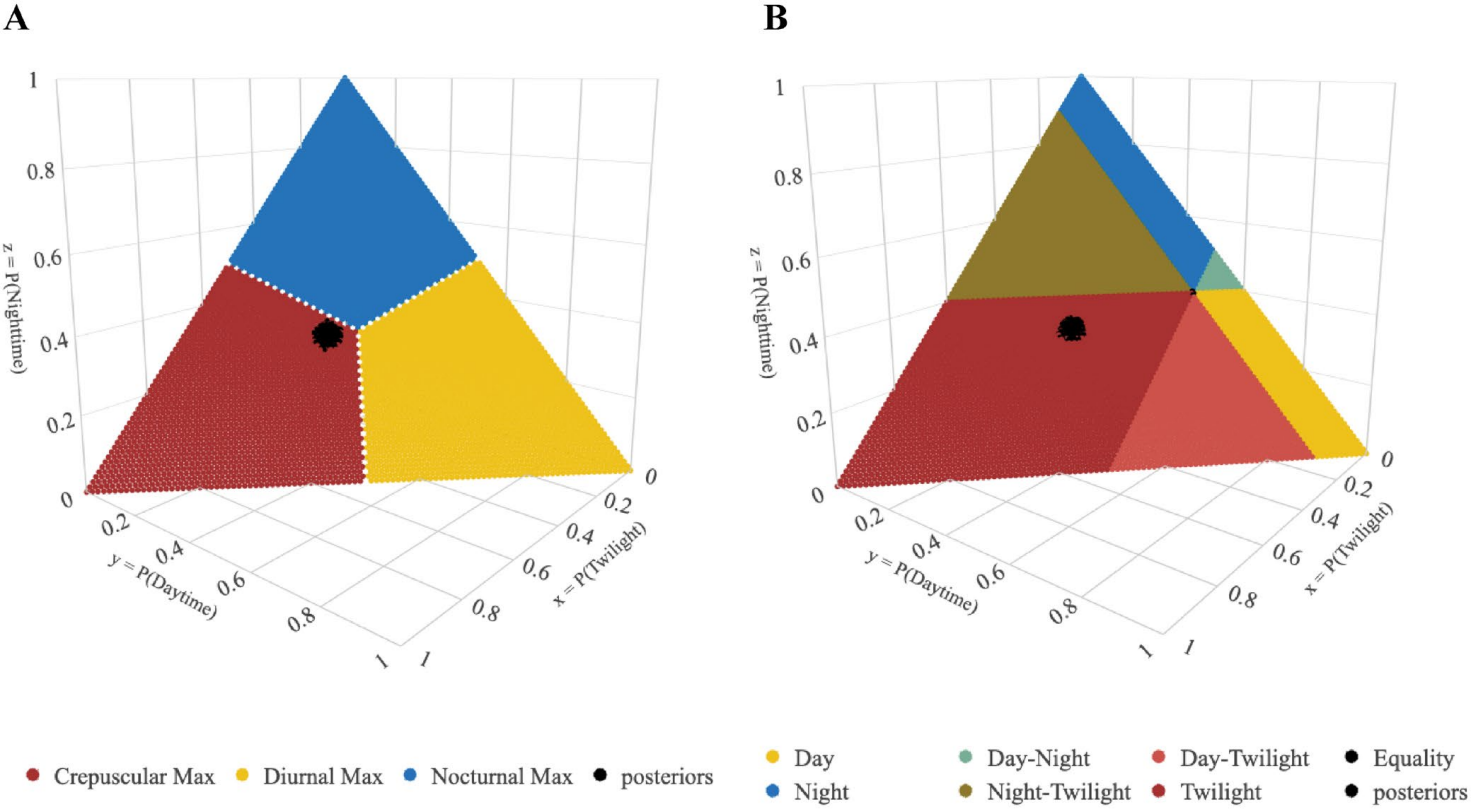
Results from the tests using the *Diel.Niche* package (Gerber et al. 2024) for (A) “Maximizing” hypotheses and (B) “Selection” hypotheses. Each plot highlights the posterior probability of the Bengal fox activity within the most‐supported time frame. The colors of each wedge correspond to the most supported activity pattern, as determined by the samples in that time segment and the results of the most supported hypothesis (e.g., Diurnal Max, Nocturnal Max, and Crepuscular Max).

The Bengal fox maximized their activity during the crepuscular period (Figure 7, posterior probability of crepuscular maximization = ~100%). Foxes had the highest proportion of total activity during the crepuscular period (median = 0.40, Bayesian confidence interval = 0.38–0.41) and exhibited strong selection for the crepuscular period compared to the crepuscular availability (average crepuscular availability = 0.11).

### 3.4 Behavioral Association at the Den Site

We were able to classify 4305 out of 4502 Bengal fox video clips into distinct behaviors as outlined in our ethogram (Table 1); the remaining 197 clips were detections where we could not determine specific behaviors. Of the classified clips, we identified 1947 distinct behavioral bouts. Of these, 80.4% detected a single type of behavior, 19.6% detected composite behaviors involving multiple individuals, with up to 3 different behaviors observed in a single clip. On average, 1.57 individuals (SD ± 0.80) were detected in these behavioral bouts (Figure 3B). We recorded a maximum of 6 foxes in a single clip and 5 foxes in 7 clips. We found no strong correlation in pairwise comparison among clip length, the number of distinct behaviors, and the maximum number of foxes detected per clip (coefficient *r* < 0.50). We detected no signs of sickness or kit mortality during the monitoring period, although we could not confirm successful dispersal of kits upon reaching maturity.

**FIGURE 8:**
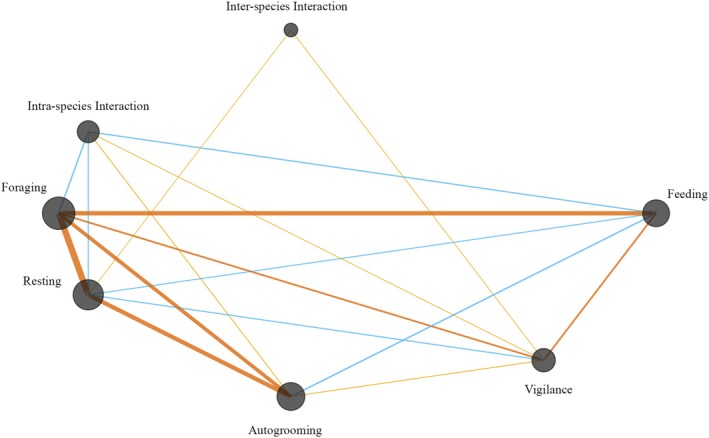
Network demonstrating co‐occurring behaviors exhibited by the Bengal fox detected in camera‐trapping at the den site in north‐western Bangladesh from 8 March 2024 to 28 May 2024 and constructed from 1947 classified behavioral bouts. Each node represents a distinct behavior. Gray circles indicate within‐node occurrence frequency. The thickness and color of the line correspond to edge weights, indicating the frequency of pairwise co‐occurrences (dark orange, high; light orange, medium; blue, low). Only behaviors with ≥ 10 co‐occurrences were included.

To visualize the structure of behavioral co‐occurrence at the den site, we constructed a weighted, undirected network of 10 behaviors (Table S3). We only retained behaviors with ≥ 10 co‐occurrences in the final network, which exhibited significant modularity (*Q* = 0.24, *p* < 0.0001), indicating the presence of distinct clusters of behavior (Figure 8). Behaviors such as feeding, foraging, resting, and autogrooming were centrally connected and showed high frequencies of co‐occurrence with multiple other behaviors. In contrast, inter‐species interaction remained weakly connected. Strong within‐behavior repetition was observed in nodes like foraging (*n* = 1185), resting (*n* = 634), feeding (*n* = 519), and autogrooming (*n* = 381), suggesting routine, non‐random behavioral patterns (Table S3).

In total, 540 video clips documented feeding behavior at the den site, evaluated across the full classified clip set. In 55% of these clips, we could not identify the prey items. In the rest, we observed foxes feeding on insects (19%, *n* = 97), birds (18%, *n* = 91), and rodents (8%, *n* = 46). Foxes exhibited distinct feeding behavior on termite swarms in 16 clips (Table S4). In one clip, we noticed foxes feeding on two items: an unidentified rodent and a bird species. The mean duration of feeding clips was short, ranging from 6.6 (termite swarm) to 12.68 s (insects).

We documented intraspecific interactions in 425 clips, with the most frequent behaviors being non‐territorial playing (*n* = 327), greeting (*n* = 50), and allogrooming (*n* = 21), while rarer behaviors included mounting (*n* = 13) and nursing (*n* = 5) (Table S4). Mean durations were 20 s, with some composite behaviors (e.g., allogrooming and playing) lasting longer than single behaviors.

Interspecific interactions were less frequent (*n* = 52), largely involving Bengal monitors (chasing, *n* = 16; stand‐offs, *n* = 31; flight, *n* = 2), and at most 2 individual Bengal monitors. The other 3 clips detected flight responses to human‐induced stimuli. These events were also brief, typically lasting under 15 s (Table S4).

## 4 Discussion

The study provides the first comprehensive quantitative account of den site behavior in the Bengal fox from Bangladesh, extending the geographic scope of behavioral data beyond existing accounts (see Vanak and Gompper 2007; Vanak et al. 2008; Punjabi et al. 2013). Although our observation utilized video segments from continuous camera trapping at a single natal den over approximately 11.5 weeks, we were able to find general support for our hypotheses. Specifically, den attendance concentrated during pup‐rearing (peak mid‐April), and foxes remained primarily crepuscular with moderate nocturnal activity. Researcher visits suppressed same‐day den activity, while interactions with Bengal monitors and humans were common yet temporally segregated. Ultimately, our observations offer empirical insights into behavioral flexibility in anthropogenic landscapes, addressing gaps in non‐apex carnivore ecology (Srivathsa et al. 2022).

The high number of fox detections (*n* = 4502) over a relatively short period reflects strong den‐site fidelity, as reported in other den‐obligate canids (Johnsingh 1978; Castelló 2018; Allen and Moll 2023; Allen and Kritzer 2023). Seasonal variation in activity, with a peak in mid‐April followed by a sharp decline in May, closely matches the species' reported reproductive cycle (Johnsingh 1978; Kumara and Singh 2012; Niraula et al. 2020). The declining detection rate likely corresponds to a gradual shift in parental investment as kits mature—for example, Johnsingh (1978) noted ‘deserted appearance’ of Bengal fox dens following a breeding cycle. Similar transitions in den use were reported in the gray fox (*Urocyon cinereoargenteus*; Allen and Kritzer 2023) and red fox (*Vulpus vulpes*; Allen and Moll 2023). The seasonal cycle of den‐site appearance and its usage pattern by foxes warrant caution when counting dens as active or inactive in density estimates. An apparently inactive den may still serve in later reproductive cycles, as evidenced by repeated reuse in foxes (Allen and Moll 2023; Allen and Kritzer 2023). Our GAM results align with the trend in fox detection rate, identifying Julian day as the strongest predictor of variations in fox activity, reflecting underlying seasonal effects (Tables 2 and 3). The lorelogram provided complementary mechanistic insight, highlighting a prolonged temporal dependency structure at the den site. Foxes' movement exhibited strong short‐term correlation for about 2 h following an initial detection. Session‐level lorelograms extended this to 168–305 min, while non‐convergence in two sessions reflects reduced activity periods rather than methodological limitations (Figure 5, Figure S1).

Research access had a measurable, same‐day suppressive effect on den activity, while incidental human movement recorded by cameras showed less effect (Table 3, Figure 4). Unlike coyotes ( *Canis latrans* ; Séquin et al. 2003), foxes showed no signs of trap shyness or behavioral suppression from human disturbance. However, the reduction in its activity (~60%) on the days we serviced the camera trap indicates that even minimal researcher presence can impact behavior in wildlife. This supports using predictive models and techniques like lorelograms to quantify observer effects (see Iannarilli et al. 2019) and improve the accuracy of behavioral inference. The contrast between research visits and routine human activity suggests that predictability, proximity, and sensory cues associated with researcher presence may be more disruptive than ambient human traffic at this site. These observations posit that minimizing den visits and standardizing camera‐trap check timing may help reduce the influence of researcher‐induced disturbance in den‐site behavioral studies.

We observed a distinct crepuscular activity pattern and strong selection for the available crepuscular period in the Bengal fox at the den site (Figures 6 and 7). This contrasts with the bimodal diurnal pattern linked to mid‐day foraging reported from Rajasthan, India (Dookia et al. 2012), and we found no evidence for a daytime preference (Figure 6). The moderate levels of nocturnal activity at the den site suggest that foxes may have been foraging during nighttime and making repeated forays to the den. These observations align with the Bengal fox biology and the central place foraging strategy typical of denning foxes (Johnsingh 1978; Gompper and Vanak 2006; Allen and Moll 2023; Allen and Kritzer 2023). But fox activity appeared temporally segregated from potential threats such as golden jackals, free‐ranging dogs, and humans (Figure 6; see Punjabi et al. 2013). The combination of central place foraging and distinct crepuscular–nocturnal activity may help the Bengal fox in avoiding potential threats. A preference for open habitats may further facilitate high visibility and early detection of threats (Figure 2; see Tannerfeldt et al. 2003).

However, these findings require a careful interpretation. First, we did not examine predictors at the home‐range scale (can be up to ~3 sq. km in males; see Vanak and Gompper 2010) or their influence on den preference. Second, both free‐ranging dogs and jackals kill fox kits (Johnsingh 1978; Vanak and Gompper 2009; Punjabi et al. 2013), and behavioral shifts in response to jackal presence are known in foxes (Scheinin et al. 2006). Moreover, although foxes maintain a specialized dietary niche (Vanak and Gompper 2009), they are often perceived as threats to poultry, which may be attributable to jackals (see Faraz et al. 2019). Road mortality is another concern (Desai et al. 2023), yet den placements close to roads are common (Punjabi et al. 2013; Akter et al. 2023). The trade‐offs of selecting roadside dens require a mechanistic assessment, and predictors of breeding success and site persistence should be evaluated across a higher ecological gradient.

While we documented 447 fox–monitor co‐detections at the den, overall diel overlap was low—potentially indicating fine‐scale, context‐dependent encounters rather than broad temporal co‐use. The den‐site interaction between Bengal foxes and Bengal monitors is a poorly understood and rarely documented dynamic, with observations only existing from Andhra Pradesh and Gujarat, India (Manakadan and Rahmani 2000; Desai et al. 2021). We noted that these interspecific interactions were usually brief (typically < 15 s), but foxes actively stood their ground, chased monitors, rarely exhibiting a flight response. This wariness is consistent with reports of monitor predation on fox kits (Manakadan and Rahmani 2000). While we observed no kleptoparasitism and could not identify whether the same individuals were making repeated visits, these medium‐sized monitors likely visited dens for leftover food or reuse. These monitors' breeding season (April–October, see Daniel 1983) overlaps with our survey period (March–May).

Our continuous camera trapping at the natal den generated an extensive and fine‐scale behavioral dataset not previously recorded for the species. High modularity suggests structured, routine den‐site activity (including vigilance, foraging, and resting) punctuated by bouts of play, grooming, and greetings. In contrast, isolated nodes emphasized rare and unpredictable behaviors that are likely context‐dependent (Figure 8). Although uncommon in wild canid research, network‐based behavioral analyses (see DeGregorio et al. 2022) offer insights into behavior sequencing in long‐term den‐use scenarios. Intra‐species interactions ranged from affiliative behaviors like allogrooming and greeting to playful interactions and nursing. While Johnsingh (1978) noted kit shyness near human settlements, we found non‐territorial chases to be the most frequent intra‐specific behavior (*n* = 327), possibly supporting social bonding or skill development among kits. Less frequent interactions, such as mounting or composite behaviors (e.g., allogrooming and playing), highlight a higher level of behavioral complexity in a species typically solitary beyond the breeding cycle (Gompper and Vanak 2006; Home and Jhala 2010).

Identifiable food items in feeding activities were consistent with findings from scat analyses of Bengal foxes living in similar human‐dominated landscapes. Insects were the most frequently detected (> 80%) prey in fox scats, alongside birds, rodents, and plant matter (Das et al. 2022; Akter et al. 2023). Predators with a central place foraging strategy are known to prefer heavier prey, such as rodents, during the nursing and rearing season (Orians and Pearson 1979). Home and Jhala (2009) observed a significantly higher Index of Relative Importance score for rodents in kit scats. In adult fox scats, rodents account for the largest volume in peri‐urban areas, whereas arthropods are the most dominant in semi‐arid environments (Das et al. 2022). Notably, feeding on termite swarms, a distinct behavior seen in 16 clips, resembled observations by Home and Jhala (2009). The study suggested this feeding activity as a near‐den opportunistic behavior in kits rather than adult provisioning. Reports of human‐derived foods in Bengal fox scats vary: Das et al. (2022) found polythene in Odisha, while Vanak and Gompper (2009) did not. Similarly, we detected foxes feeding on rodents in 8% of video clips but did not document feeding activity on plant matter, human‐derived food, or rare food items like snakes as observed by Desai et al. (2022).

Bengal foxes' nocturnal, elusive lifestyle, coupled with limited conservation focus (see Vanak et al. 2008) and the underuse of non‐invasive tools, often hinders understanding their ecology (see Manakadan and Rahmani 2000). Camera trapping has proven effective for documenting complex behavioral repertoires, as shown here in studies on red and gray foxes, and several other denning species (Caravaggi et al. 2017; Allen and Moll 2023; Allen and Kritzer 2023). Molecular tools such as non‐invasive scat DNA analysis would allow behavioral and ecological inferences to be scaled to the population level (see Home and Jhala 2009; Das et al. 2022). Simultaneous and longitudinal multi‐den monitoring during the same breeding season would help understand site‐level effects and population structure (see Home and Jhala 2010). Telemetry studies would further clarify the relationship between central place foraging behavior and home‐range use, an aspect rarely studied (see Vanak and Gompper 2010).

For a species that lives in shared landscapes and is declining across much of its range (Kumara and Singh 2012; Punjabi et al. 2013; Khan 2015; Jhala 2016; Akter et al. 2023), it is challenging to design effective conservation strategies. We propose that local‐level conservation planning for Bengal foxes should consider conserving non‐protected areas, such as village groves and natural fallow land—habitats shared by similarly challenged wildlife. In Bangladesh, these measures should further be coupled with den awareness, den‐based density counts and an update of the country‐wide range for Bengal foxes—an aspect that has never been addressed.

Den‐site behavior is inherently context‐dependent, shaped by local predator assemblages, human disturbance regimes, habitat structure, and the reproductive stage of the resident pair. Observations from a single den cannot capture the full range of behavioral variation within the species, warranting caution with our extrapolations. Nevertheless, given the rarity of the Bengal fox and the near‐complete absence of published fine‐scale den‐site behavior data, even single‐den observations provide an empirical foundation for future studies. Our approach thus highlights the value of integrating fine‐scale modeling with behavioral data and provides a reproducible framework for studying similar cryptic, declining species living close to human settlements but falling outside conservation investments.

## Author Contributions

**Muntasir Akash:** conceptualization (lead), data curation (supporting), formal analysis (lead), investigation (equal), methodology (lead), project administration (lead), resources (lead), software (equal), supervision (equal), visualization (equal), writing – original draft (equal), writing – review and editing (supporting). **Md. Rokonuzzaman:** investigation (equal), methodology (supporting), project administration (equal). **Sultan Ahmed:** investigation (supporting), methodology (supporting). **Mohammad Samiul Alam:** data curation (lead), investigation (supporting), methodology (supporting), software (supporting). **Maximilian L. Allen:** supervision (lead), validation (lead), visualization (supporting), writing – original draft (supporting), writing – review and editing (lead).

## Funding

The research was a self‐funded endeavor.

## Ethics Statement

This manuscript does not include human or animal handling research.

## Conflicts of Interest

The authors declare no conflicts of interest.

## Supporting information


**Table S1:** ece373371‐sup‐0001‐Supinfo.docx.

## Data Availability

All files (CSVs, *R scripts, RProj*, and *RData*) used to carry out analyses associated with this manuscript are available at: https://github.com/lynx025/Bengal‐Fox‐denning‐behavior.git.
