# Supplementary material for: Den‐Site Behavior of Bengal Foxes (Vulpes bengalensis) Reveals Persistent Use, Social Interactions, and Coexistence in Shared Spaces
Source: Ecol Evol. 2026 Apr 2;16(4):e73371. doi: 10.1002/ece3.73371 (PMC13045414; doi:10.1002/ece3.73371)
Supplement: Supplementary file 1 — Table S1: ece373371‐sup‐0001‐Supinfo.docx. [file ECE3-16-e73371-s001.docx]

**SUPPLEMENTARY FILE**

***Supplementary Table 1***. Description, justification and relevant a priori predictions (positive or negative) for covariates used to predict the den site activity of the Bengal fox in Northern Bangladesh with a generalized additive modeling (GAM) approach and negative binomial structure. The Bengal fox detections per active camera trap day were used as a proxy of the fox activity level at the den site captured in camera trapping from 08 March 2024 to 28 May 2024

| **Covariates** | **Type** | | **Justification** | ***a priori* prediction** |
| --- | --- | --- | --- | --- |
| Julian Day | Numerical | The Bengal fox is an obligate natal denning species. In non-breeding period, dens appear to have a deserted appearance, indicating a low extent of activity around the den site (see Johnsingh 1978, Manakadan and Rahmani 2000, Punjabi et al. 2013). | | negative |
| Camera trap check | Binary | Presence of camera-trap is reported to develop camera trap shyness or avoidance behavior in carnivore mammals; thus, frequent checks may impact natural denning activity in the Bengal fox (see Tourani et al. 2020, Mackaughan et al. 2023). | | negative |
| Days since last check | Numerical | Presence of camera-trap is reported to develop camera trap shyness or avoidance behavior in carnivore mammals; thus, in case of such impact, activity of foxes will show a decreasing trend (i.e., low detectability) in the following days after a check occurred and will be lower with each subsequent checks (see Tourani et al. 2020, Mackaughan et al. 2023) | | negative |
| Bengal monitor detections | Numerical | Presence of the Bengal monitor around the Bengal fox den site is noted and reported as a threat due to reported instances of predation on fox pups by monitors (see Manakadan and Rahmani 2000, Desai et al. 2021). | | negative |
| Human movement detections | Numerical | Denning behavior of the Bengal fox are affected by anthropogenic activities (see Gompper and Vanak 2006, Desai et al. 2023). | | negative |

***Supplementary Table 2***. Comparison of top GAM models to predict the den site activity of the Bengal fox in Northern Bangladesh. The Bengal fox detections per active camera trap day were used as a proxy of the fox activity level at the den site captured in camera trapping from 08 March 2024 to 28 May 2024

| Models | ΔAIC | wAIC | AIC | BIC | Adj. R-squared | RMSE |
| --- | --- | --- | --- | --- | --- | --- |
| s(Julian day) + s(days since last check) + camera trap check + Bengal monitor detection + Human movement detection | 0.0 | 0.44 | 546.2 | 573.9 | 0.5 | 54.7 |
| s(Julian day) | 1.9 | 0.17 | 548.1 | 560.6 | 0.5 | 62.6 |
| s(Julian day) + s(days since last check) + Bengal monitor detection + Human movement detection | 3.3 | 0.08 | 549.5 | 572.4 | 0.5 | 57.0 |
| s(Julian day) + s(days since last check) + Human movement detection | 3.7 | 0.07 | 549.9 | 570.1 | 0.4 | 62.1 |
| s(Julian day) + s(days since last check) | 5.7 | 0.03 | 551.9 | 565.9 | 0.5 | 62.2 |
| s(Julian day) + s(days since last check) + Bengal monitor detection | 7.2 | 0.01 | 553.4 | 581.8 | 0.5 | 57.2 |
| ~ 1 | 71.2 | ~0.0 | 617.4 | 621.5 | 0.0 | 88.0 |

*Likelihood-ratio test statistics between the two top-ranked nested models (Analysis of Deviance Table)*

| Model 1: ~ s(Julian day) | | | | | |
| --- | --- | --- | --- | --- | --- |
| Model 2: ~ s(Julian day) + s(days since last check) + camera trap check + Bengal monitor detection + Human movement detection | | | | | |
| Model | Residual degree of freedom | Residual deviance | Degree of freedom | Deviance | *P*-value of chi-squared test |
| 1 | 52.1 | 535.9 |  |  |  |
| 2 | 44.1 | 519.4 | 8.0 | 16.5 | 0.03 |


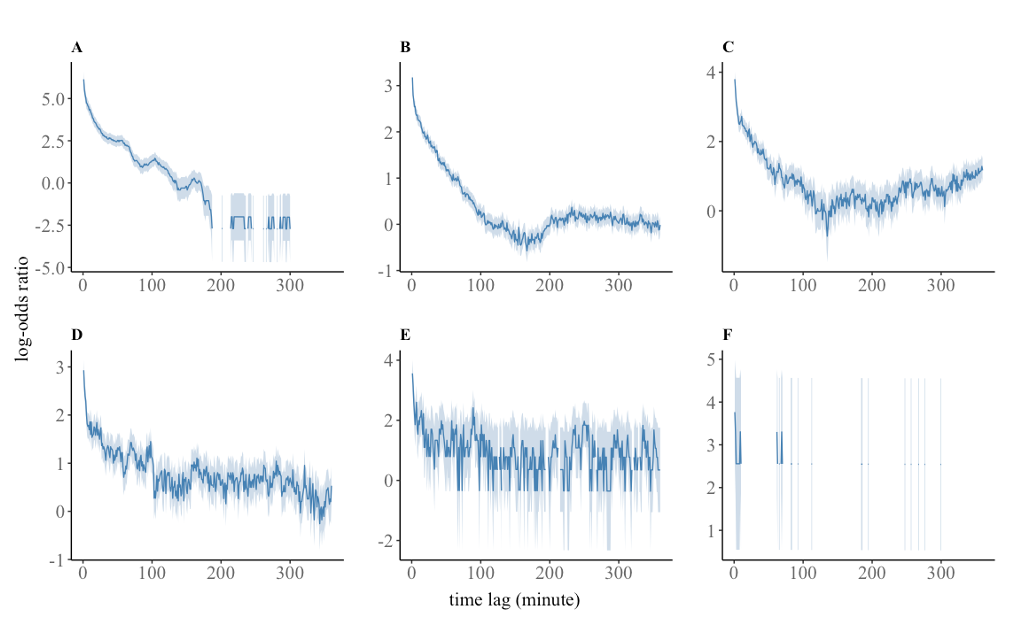


***Supplementary Figure 1.*** Estimated lorelograms and 95% confidence intervals (shaded areas) for time-lags between 0 and 360 min for Bengal fox *Vulpes bengalensis* data collected in northern Bangladesh from March 2024 to May 2024, compared across sessions divided by a round of camera-trap data checking. **A**. March 08 – March 09; **B**. March 18 – March 31; **C**. March 18 – April 09; **D**. April 19 – April 30; **E**. May 09 – May 15; and **F.** May 15 – May 28. Gaps in dates indicate camera-trap malfunctions. Gaps in estimated lorelograms (**A** and **F**) reflect time lags with too few detection pairs to reliably estimate log-odds ratios.

***Supplementary Table 3*.** Adjacency matrix showing the number of pairwise co-occurrences among 11 distinct Bengal fox activities detected in camera-trapping at the den site in northern Bangladesh between 08 March 2024 to 28 May 2024 and constructed from 1,947 classified behavioral bouts.

|  | **Feeding** | **Caching Food** | **Foraging** | **Inter-species Interaction** | **Intra-species Interaction** | **Passing** | **Defecating** | **Resting** | **Autogrooming** | **Vigilance** |
| --- | --- | --- | --- | --- | --- | --- | --- | --- | --- | --- |
| **Feeding** | 519 |  |  |  |  |  |  |  |  |  |
| **Caching Food** | 0 | 8 |  |  |  |  |  |  |  |  |
| **Foraging** | 56 | 0 | 1185 |  |  |  |  |  |  |  |
| **Inter-species Interaction** | 0 | 0 | 5 | 52 |  |  |  |  |  |  |
| **Intra-species Interaction** | 19 | 0 | 53 | 1 | 428 |  |  |  |  |  |
| **Passing** | 142 | 0 | 234 | 17 | 120 | 2035 |  |  |  |  |
| **Defecating** | 0 | 0 | 0 | 0 | 0 | 0 | 2 |  |  |  |
| **Resting** | 31 | 0 | 89 | 2 | 49 | 195 | 1 | 634 |  |  |
| **Autogrooming** | 23 | 0 | 80 | 3 | 29 | 119 | 0 | 73 | 381 |  |
| **Vigilance** | 25 | 0 | 56 | 25 | 26 | 86 | 0 | 23 | 17 | 446 |

***Supplementary Table 4*.** Summary of Bengal fox behavioral events observed in all classified video clips (n = 4,305). Table includes the number of clips, mean duration (seconds), and standard deviation (SD) for each behavior type, grouped under behavioral categories (intra-species, inter-species, feeding). Data derived without filtering for distinct behavioral bouts.

| Behavior types | No. of video clips detected | Mean duration (sec) | SD duration (sec) |
| --- | --- | --- | --- |
| **Intra-species Interaction** | | | |
| Mounting | 13 | 15.08 | 12.97 |
| Greeting | 50 | 13.12 | 9.72 |
| Greeting, Allogrooming | 6 | 38.83 | 18.87 |
| Allogrooming, Playing | 3 | 26.67 | 15.28 |
| Allogrooming | 21 | 20.57 | 19.38 |
| Allogrooming, Greeting | 1 | 10.00 | NA |
| Allogrooming, Playing | 3 | 37.33 | 20.53 |
| Nursing | 5 | 10.00 | 0.00 |
| Playing (non-territorial fights/chases) | 327 | 13.59 | 11.98 |
| **Inter-species Interaction** | | | |
| Interacting with human or human-induced disturbance/Flight | 3 | 13.33 | 5.77 |
| Interacting with monitor/Chasing | 16 | 14.06 | 5.09 |
| Interacting with monitor/Flight | 2 | 17.00 | 0.00 |
| Interacting with monitor/Stand-off | 31 | 14.55 | 6.50 |
| **Feeding** | | | |
| Insects | 81 | 12.68 | 10.24 |
| Bird | 92 | 11.12 | 9.35 |
| Bird, Rodent | 1 | 20.00 | NA |
| Rodent | 46 | 12.35 | 12.00 |
| Termite Swarm | 16 | 6.63 | 4.19 |
| Unidentified | 284 | 11.16 | 9.29 |

**References**

Desai P, Patel S, Saudhary S (2021) An interesting observation: Monitor lizard (*varanus bengalensis*) using the den of Indian fox (*Vulpes bengalensis*). Prithivya, an official newsletter of WCB research foundation and WCB research lab. 1: 4–6.

Desai P, Rabari V, Dharaiya N (2023) Are Indian foxes vulnerable in degraded habitat in North Gujarat? Taprobanica 12: 101–103.<http://dx.doi.org/10.47605/tapro.v12i2.313>

Gompper ME, Vanak AT (2006) *Vulpes bengalensis*. Mammalian Species 795: 1–5.

Johnsingh AJT (1978) Some aspects of the ecology and behaviour of the Indian fox *Vulpes bengalensis*. Journal of Bombay Natural History Society 75: 397–405.<https://biostor.org/reference/148075>

McKaughan JE, Stephens PA, Hill RA (2023) Estimating mesocarnivore abundance on commercial farmland using distance sampling with camera traps. Ecological Solutions and Evidence 4: e12229.<https://doi.org/10.1002/2688-8319.12229>

Manakadan R, Rahmani AR (2020) Population and ecology of the Indian fox *Vulpes bengalensis* at Rollapadu wildlife sanctuary, Andhra Pradesh, India. Journal of the Bombay Natural History Society 97: 3–14.<https://biostor.org/reference/151708>

Punjabi GA, Chellam R, Vanak AT (2013) Importance of native grassland habitat for den-site selection of Indian foxes in a fragmented landscape. PLoS One 8: e76410.<https://doi.org/10.1371/journal.pone.0076410>

Tourani M, Brøste EN, Bakken S, Odden J, Bischof R (2020) Sooner, closer, or longer: detectability of mesocarnivores at camera traps. Journal of Zoology 312: 259–270.<https://doi.org/10.1111/jzo.12828>
